# Clinical Characteristics and Neuroimaging Correlations in Autoimmune Encephalitis: A Report of Two Cases

**DOI:** 10.7759/cureus.54257

**Published:** 2024-02-15

**Authors:** Carla B Graos, Victor M Montalvan, Percy Torres, Camila Coboj, Fernando S Geldres

**Affiliations:** 1 Department of Neurology, Hospital Victor Lazarte Echegaray, Trujillo, PER; 2 Department of Neurology, Hospital Víctor Lazarte Echegaray, Trujillo, PER; 3 School of Medicine, Autonomous University of Durango, Durango, MEX; 4 Department of Neurosurgery, Hospital Victor Lazarte Echegaray, Trujillo, PER

**Keywords:** autoimmune, encephalopathy, autoimmune encephalitis negative serotype, antibody against the n-methyl -d receptor - aspartate (anti - nmda), autoimmune encephalitis

## Abstract

Autoimmune encephalitis is an infrequent pathological occurrence documented within our local context. When clinical suspicion arises, employing electroencephalogram and brain magnetic resonance imaging (MRI) proves valuable. However, for conclusive diagnosis confirmation, lumbar puncture for cerebrospinal fluid (CSF) analysis is indispensable. Managing this condition involves a combination of immunosuppression and, when necessary, tumor resection. We document the initial cases reported in our city, featuring two young patients without significant pre-existing conditions. Patients initially displayed behavioral alterations progressing to altered consciousness, febrile peaks, and challenging epileptic status, requiring intensive care and mechanical ventilation. The diagnosis was made based on MRI and anti-N-methyl-D-aspartate (anti-NMDA) antibodies. Treatment involved intravenous (IV) immunoglobulins, plasmapheresis, and corticosteroids. After discharge, both had cognitive sequelae. Autoimmune encephalitis is a pathological condition that still lacks thorough exploration and reporting. It predominantly affects young patients without a history of acute psychiatric symptoms, necessitating consideration when behavioral alterations emerge. The challenges faced by small cities, including a shortage of technical resources, further impede the timely and precise diagnosis of this intricate condition.

## Introduction

Autoimmune encephalitis involves the inflammation of brain tissue triggered by immunological factors, including antibodies or cellular immunity acting against antigens found in the brain parenchyma, meninges, and cerebral vessels [1,2].

Autoimmune encephalitis exhibits a prevalence of 13.7 cases per 100,000 people annually, with anti-N-methyl-D-aspartate (NMDA) encephalitis being the most common cause [3]. In the United States, an estimated 20,000 cases of encephalitis occur yearly, with 50% remaining inconclusive for infectious causes, indicating a potential autoimmune link [4,5]. In Peru, only a few cases have been reported [2,6,7], underscoring the vital need for ongoing dissemination of information regarding the diagnosis and treatment protocols among institutions.

We introduce the initial two cases reported in the literature from our city, featuring two young patients, one with a negative serotype of autoimmune encephalitis and the other with an anti-NMDA serotype. Both presented with initial behavioral alterations, followed by an altered level of consciousness, febrile peaks, and a challenging-to-control epileptic status.

## Case presentation

Case 1

History and Examination

A 16-year-old male, devoid of any pathological or family history, presented with a week-long history of fever, general malaise, and cough. Despite a negative pharyngeal swab antibody study for SARS-CoV-2, he received symptomatic treatment. The patient was admitted to the emergency room following a generalized tonic-clonic seizure of unknown onset. Postictally, he exhibited behavioral alteration for 15 minutes, followed by bradypsychia, retrograde and semantic amnesia, and acalculia persisting for 45 minutes. Neurological evaluation revealed an impairment of higher brain functions such as higher-order thinking, information processing, and reasoning, with no cranial nerve abnormalities, motor deficits, or signs of meningeal involvement. During his hospitalization, the patient manifested unilateral brachio-facial clonic seizures originating in the hand and progressing to the face. These were accompanied by generalized tonic-clonic seizures featuring ocular retroversion and postictal drowsiness lasting around 20 minutes. Basic serum analysis and cerebrospinal fluid (CSF) study showed a slight cellularity increase with normal glucosa and proteins which favored the diagnosis of a viral infection, prompting the initiation of intravenous (IV) phenytoin (100 mg every 8 hours) and acyclovir (10 mg/kg every 8 hours). Despite this treatment for three days, there was a notable escalation in the frequency of epileptic seizures, peaking at 3 to 4 times per day. The situation progressed to episodes of generalized tonic-clonic status epilepticus, unresponsive to phenytoin and levetiracetam at loading doses. Suspecting autoimmune encephalitis due to the evolving condition, methylprednisolone pulses were initiated after defocalization, administered at a dose of 1,000 mg IV every 24 hours for five days. The management of status epilepticus in the intensive care unit involved high doses of benzodiazepines, resulting in an appropriate response.

Diagnostic Evaluation

Comprehensive laboratory tests were conducted, revealing a complete blood count with leukocytosis (14.530), hemoglobin at 16 mg/dl, and platelets at 214,000. The liver profile, coagulation profile, arterial blood gas, and electrolyte analysis all returned within normal ranges. The blood group was identified as O+, while the urine test and culture were normal and negative, respectively. The extractable nuclear antigen test, anti-neutrophil cytoplasmic antibodies (ANCA), myeloperoxidase, virological panel, and fungal and bacterial cultures were all negative. Tumor markers remained within normal values.

The CSF analysis (Table 1) showed just a slight pleocytosis, the immunological panel (Table 2) further revealed negative results for anti-NMDA, α-amino-3-hydroxy-5-methyl-4-isoxazolepropionic acid receptor (AMPA), γ-aminobutyric acid sub-type B (GABA B), leucine-rich glioma-inactivated 1 (LGI1) and contactin-associated protein-2 (CASPR2).

**Table 1:** Main characteristics of cerebrospinal fluid analysis ADA, adenosine deaminase; BK, bacilloscopy.

Patient	Appearance	Proteins	Cells	Glucose	Pressure	ADA	Gram stain	Chinese ink	BK
Case 1	Turbid	45 mg/dl	15 cells/ mm^3^	50 mg/dl	80 mmH_2_O	3.18 U/l	Negative	Negative	Negative
Case 2	Mild turbid	39 mg/dl	3 cells/mm^3^	65 mg/dl	90 mmH_2_O	2 U/l	Negative	Negative	Negative

**Table 2:** Immunological panel in CSF CSF, cerebrospinal fluid; NMDA, N-methyl-D-aspartate; AMPA, α-amino-3-hydroxy-5-methyl-4-isoxazolepropionic acid; GABA B, γ-aminobutyric acid sub-type B; LGI1, leucine-rich glioma-inactivated 1.

Patient	NMDA	AMPA 1-2	GABA B	CASPR2	LGI1
Case 1	Negative	Negative	Negative	Negative	Negative
Case 2	Positive	Negative	Negative	Negative	Negative

The thymus and testicular ultrasound results were within normal limits, and examinations of the brain, abdominopelvic tomography, and brain magnetic resonance imaging (MRI) revealed no abnormalities. The conclusive diagnosis pointed toward seronegative autoimmune encephalitis.

Evolution

The patient underwent an extensive 55-day stay in the ICU, receiving targeted treatments including immunoglobulin at a dosage of 0.4 mg/kg IV for five days (administered in two cycles). A month later, plasmapheresis was conducted over five sessions. The prolonged hospitalization led to several complications, including respiratory failure necessitating mechanical ventilation, a tracheostomy, urinary tract infection, nosocomial pneumonia, and polyneuropathy in the critically ill patient.

Upon discharge from the ICU, the patient exhibited signs of disorientation in time and partial spatial confusion, with a Mini-Mental State Examination score of 25 points. Further assessment by the psychology service involved the NEUROPSI test (Table 3) to gauge the extent of cognitive sequelae, revealing a severe impairment.

**Table 3:** Main neuropsychological characteristics of patients - NEUROPSI test

Neuropsychological test: NEUROPSI	Case 1	Case 2
Concentration attention	21/26	16/26
Evocation	8/30	5/30
Lecture	0/3	0/3
Codification	11/18	9/18
Orientation	6/6	5/6
Language	17/26	15/26
Handwriting	1/2	1/2
Executive functions	13/18	11/18
Final score	77/129	62/129
Conclusion	Severe damage	Severe damage

Following his hospital discharge, he faced challenges in autonomously carrying out fundamental activities, necessitating assistance for mobility. Notable physical consequences encompassed left temporal hemianopsia and quadriparesis, particularly affecting the distal regions, resulting in pronounced muscle atrophy. The patient was readmitted a month later for refractory status epilepticus and was subsequently referred to a National Neurology Institute for specialized management under the guidance of epileptologists.

Case 2

History and Examination

A 31-year-old female architect, with a medical history of untreated polycystic ovary syndrome and treated gastritis, presents a one-month history of illness. Initially marked by challenges in comprehending written language, bradypsychia, difficulty understanding spoken language, and paresthesias in the right hemibody, her condition progressed to include insomnia, auditory hallucinations ("hearing music and alarm sounds without sound stimulation"), dysarthria, and echolalia. Upon psychiatric evaluation, the patient was diagnosed with acute stress and an anxious-anankastic personality. The prescribed treatment regimen consisted of sertraline, risperidone, and clonazepam. At her residence, persistent echolalia and drowsiness characterize the patient's state, marked by a notable lack of communication with family members. Subsequent evaluation by a private psychiatrist led to a revised diagnosis of an acute psychotic episode and obsessive-compulsive disorder. The previous medication was discontinued, and a new regimen was initiated, including olanzapine, paroxetine, and biperiden.

Despite these changes, the patient's symptoms escalated, encompassing insomnia, prolonged complex motor automatisms (such as gestural actions and object manipulation lasting over 20 minutes), and a disconnection from her immediate environment. Disturbingly, these episodes were occasionally accompanied by urinary incontinence. Moreover, the emergence of unmotivated sardonic laughter, shouting, and disorganized, uninhibited behavior prompted a shift in medication to levomepromazine and clonazepam at maximum doses, yet clinical improvement remained elusive. Further complicating matters, additional movements manifested, including chewing-like motions, jumping, and repetitive pelvic movements. Upon reassessment by another psychiatrist, the patient received diagnoses of dissociative and extrapyramidal syndrome, along with suspicions of neuroleptic malignancy.

Diagnostic Evaluation

Upon admission to the emergency room, the patient underwent evaluation by the neurology service, revealing spontaneous eye opening, a lack of connection with the environment, non-compliance with orders, absence of verbal expression, and an inability to assess higher brain functions. The neurological physical examination yielded normal results. The patient exhibited subacute alterations in the level of consciousness, stereotyped extremity movements, psychomotor agitation, and disorganized behavior, and was on multiple medications. Given the inadequate response to neuroleptics and considering the age factor, suspicion arose regarding autoimmune encephalitis.

Comprehensive general tests, including blood count, thyroid profile, lipid profile, and urea and creatinine levels, all fell within normal limits, except for vitamin B12 levels, indicating a mild deficiency at 163 pg/ml (normal range: 200-1000). Additionally, the extractable nuclear antigen antibody profile was negative, and tests for ANCA and myeloperoxidase were also negative. The virological panel and fungal and bacterial cultures returned negative results. Tumor markers remained within normal values. Serum antibody determination proved negative. CSF analysis was normal (Table 1). While in the immunological panel (Table 2) anti-NMDA antibodies were detected, indicating a positive result.

**Figure 1:**
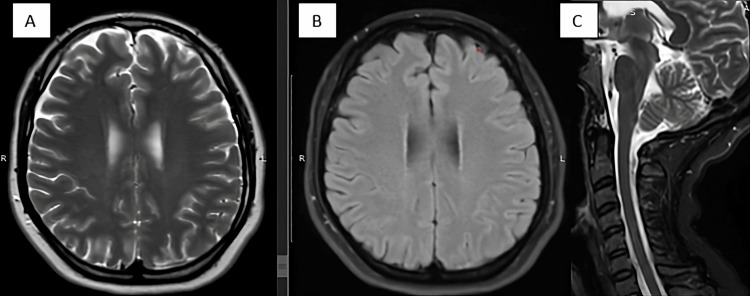
Initial brain and spine MRI A, B: Axial T2 and axial FLAIR show no structural injuries. C: STIR – cervical spine cord shows no lesions. MRI, magnetic resonance imaging; FLAIR, fluid-attenuated inversion recovery; STIR, short tau inversion recovery.

**Figure 2:**
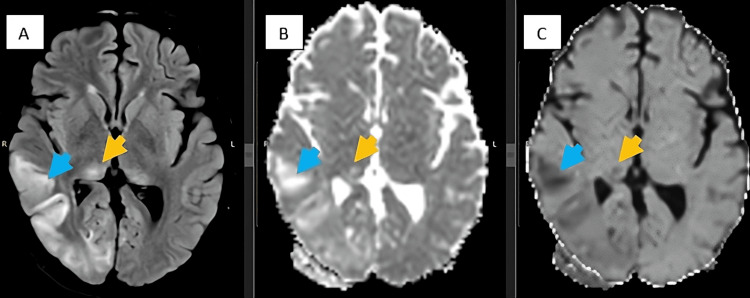
Three-month follow-up brain MRI A: Axial FLAIR shows a right temporo-occipital cortical hyperintense lesion (blue arrow) and bilateral pulvinar nucleus (yellow arrow). B, C: injuries restrict diffusion. FLAIR, fluid-attenuated inversion recovery.

Ultrasound of the thymus yielded normal results, and brain tomography revealed findings within the normal range. Contrast-enhanced thoraco-abdomino-pelvic tomography identified a cyst in the right ovary, with no evidence of masses or tumors. Initial brain MRI displayed normal results (Figure 1), but a follow-up after three months (Figure 2) revealed hyperintense cortical lesions in T2 and FLAIR sequences, demonstrating restricted diffusion in the right parieto-occipital lobe and bilateral pulvinar nuclei. The conclusive diagnosis pointed to anti-NMDA autoimmune encephalitis.

Evolution

Throughout the hospitalization, the patient experienced peaks of fever, accompanied by persistent generalized tonic-clonic seizures. These seizures were characterized by an absence of consciousness recovery, ocular retroversion, and sustained sphincter contraction unresponsive to diazepam or IV phenytoin. Despite treatment with antiepileptic drugs, specifically valproic acid (500 mg PO/12 hours) and levetiracetam (1000 mg PO/12 hours), the status epilepticus persisted. Consequently, the patient was transferred to the ICU, where she remained for two months. During this ICU stay, the patient underwent treatment with immunoglobulin at a dosage of 0.4 mg/kg IV for five days, followed by plasmapheresis consisting of seven sessions conducted a month later.

After an extended hospital stay, she faced various complications, including respiratory failure necessitating mechanical ventilation, a tracheostomy, urinary tract infection, distributive shock, nosocomial pneumonia, digestive bleeding, and gastroenteritis. The treatment protocol involved the administration of valproic acid at 500 mg PO/8 h, along with melatonin and zolpidem to ensure effective control of sleep patterns, while successfully weaning off ventilation and managing epileptic seizures. As her consciousness gradually returned, she regained the ability to perform basic activities independently. However, lingering symptoms included bradypsychia and mild cognitive impairment. Discharge from the ICU revealed some disorientation in time and partial spatial confusion, as indicated by a Mini-Mental Test score of 24 points, and the neuropsychological test revealed severe damage (Table 3).

Upon hospital discharge, she achieved independence in basic activities, though she continues to experience bradypsychia and mild cognitive impairment. Despite these challenges, her clinical progress has been favorable and gradual. Although there have been minor setbacks in neuropsychological evaluations, she adeptly handles daily activities autonomously and has aspirations to resume work. She is currently maintaining a regimen of lacosamide at 50 mg PO bid and azathioprine at 50 mg qid. She now attends regular check-ups to monitor her condition.

## Discussion

Autoimmune encephalitis is reported in 1-4% of patients with encephalitis of unknown cause, predominantly affecting young adults, particularly women. However, cases have also been reported in pediatric populations [5]. The etiology is unknown. However, the production of autoantibodies may be related to a neoplastic syndrome which is more prevalent in young adults but is rarely found in the infant population. In 85% of the cases, the presentation of adults is clinically unspecific, unlike in pediatric cases where the clinical presentation is predominantly neurological [8].

The clinical presentation of this condition can manifest through various syndromes, posing a challenge for early recognition and subsequently leading to a diagnosis challenge. Nevertheless, distinct phases of the disease have been identified: prodromal period, intermediate, and late period [9].

Examining the presented cases, we note a correlation between their course and the documented literature. In Case 1, the prodromal period exhibited symptoms similar to viral infections, while in Case 2, the manifestations were less defined, perhaps influenced by the patient's solitary living situation. Both cases converged in an intermediate phase marked by psychiatric symptoms such as irritability, behavioral issues, and sleep disturbances. Notably, neurological symptoms were absent during this stage, contributing to misdiagnosis in a significant percentage of cases (72-84%) [2]. Consequently, it is common for patients to initially seek evaluation from psychiatrists rather than neurologists due to this distinctive clinical presentation.

In the final phase, both patients showed neurological symptoms. Case 1 displayed alterations in consciousness, orofacial dyskinesias, and hypoventilation linked with refractory epilepsy, necessitating intubation and mechanical ventilation. Meanwhile, Case 2 presented altered consciousness, movement disorders, dystonic posture, aphasia/dysarthria, and a rapid progression of psychosis despite therapy. Additionally, autonomic instability and refractory epilepsy mandated intubation and mechanical ventilation. Notably, epileptic seizures, prevalent in 80% of cases according to the literature [9], marked our patients' disease evolution.

In Case 1, despite the absence of a recognized antigen in serum or CSF, the presence of symptoms such as encephalitis with memory deficit, refractory epileptic seizures, and drug-resistant status epilepticus raised suspicion of potential associations with antigens such as AMPA receptor or GABA B. Comprehensive investigations ruled out occult neoplasm in the lung, thymus, or testicles. Unfortunately, due to limited financial resources, an extensive autoimmune panel study couldn't be conducted, leading to the classification of the case as serotype negative [10]. Contrastingly, Case 2 exhibited positive anti-NMDA encephalitis in the CSF, but no ovarian teratoma or other tumors were detected elsewhere. It is crucial to underscore that in instances where neoplastic origin is suspected, especially in high-risk syndromes or cases involving specific antibodies as seen in Case 2, prompt neoplasm screening is imperative. This urgency stems from the fact that neurological syndromes often precede tumors in about two-thirds of cases. Commonly associated tumors include those of the lung, breast, lymphoma, or thymoma [7]. Given the significant association with paraneoplastic (40%-58% of cases), screening should be reiterated within 3-6 months [10].

In low-resource settings, diagnosing rare illnesses is particularly challenging, especially when specific tests are required. Often, clinicians rely on clinical judgment or make diagnoses by exclusion due to limited resources. In many cases, definitive diagnoses of neurological diseases such as autoimmune encephalitis, epilepsy, and degenerative conditions necessitate studies that are unavailable locally. Case 1 underwent a clinical diagnosis, with seronegative markers. In a different context, if available, a comprehensive immunological profile, a PET scan, or even a brain biopsy could be employed to obtain a definitive diagnosis.

The prevailing therapeutic approach involves a sequential two-line immunotherapy strategy: initiating with steroids (methylprednisolone 1 g IV daily for five days) and IV immunoglobulin (IV Ig mg/kg body weight per day for five days), often complemented by plasma exchange. If there is no favorable response within 2-3 weeks after starting the first line, second-line treatment is recommended. This may involve rituximab (375 mg/m^2^ weekly for four weeks or 1 g twice separated by two weeks) and/or cyclophosphamide (750 mg/m^2^ monthly for four to six months, depending on the progression) [11].

Both patients, Case 1 requiring 55 days of intensive care and Case 2 requiring 77 days, underwent specific treatments tailored to their conditions. In Case 1, treatment included immunoglobulin (0.4 mg/kg IV for five days in two cycles) and plasmapheresis (five sessions). Case 2 received methylprednisolone (1 g IV once), immunoglobulin (0.4 mg/kg IV for five days in two cycles), and plasmapheresis (seven sessions). While patients did receive treatment, there is a possibility that they could have benefited from newer therapeutic strategies like rituximab or cyclophosphamide. Unfortunately, these options pose challenges due to their high cost and limited accessibility in our country. Moreover, they are not authorized to manage this disease within our institution.

Upon discharge, Case 1 struggled to perform basic activities independently, requiring support for mobility. Contrastingly, Case 2 regained independence in basic activities, though bradyphrenia and mild cognitive impairment persisted. The literature suggests that 75% of patients experience a favorable response with minimal or no sequelae [1]. The recovery process typically unfolds in the reverse sequence of symptom onset, with mortality hovering around 4% and relapses occurring in 20-25% of patients [9]. Case 2 aligns with this pattern, demonstrating a progressive clinical improvement after discharge. Despite minor hitches in neuropsychological evaluations, she autonomously manages daily activities and plans to resume work.

## Conclusions

In summary, autoimmune encephalitis stands as a relatively understudied and underreported medical phenomenon, necessitating institutions to actively advocate for enhanced exploration and documentation of such cases. It is imperative to contemplate the potential presence of autoimmune encephalitis, particularly in young patients displaying profound behavioral alterations without a history of acute psychiatric manifestations.

Our study strongly underscores the need for systematic screening for autoimmunity in cases of subacute encephalopathy lacking a clear etiology. While a conclusive diagnosis often relies on demonstrating neuroglial antibodies, the absence of such antibodies does not definitively negate an autoimmune origin. In low-resource settings, we strongly recommend proactively initiating immunomodulatory therapy based on clinical suspicion, even in the absence of confirmatory results. This approach aims to prevent avoidable delays and minimize the risk of neurological sequelae.

## References

[1] Collao-Parra JP, Romero-Urra C, Delgado-Derio C (2018). [Autoimmune encephalitis. A review]. Rev Med Chil.

[2] Palomino H, Segura D, Quispe D (2019). Encefalitis autoinmune mediada por anticuerpos contra el receptor N-metil-D-aspartato: Reporte de cuatro casos en Perú. Rev Peru Med Exp Salud Publica.

[3] Bazalar G, Azañero J, Piscoya T, Chambi L, Soto A (2023). Encefalitis autoinmune por anticuerpos NMDA-R en tiempos del COVID-19. Rev Soc Peru Med Interna.

[4] García-Beristáin JC, Barragán-Pérez E, Choperena-Rodríguez R, Reyes-Cruz G (2017). Encefalitis autoinmune en pediatría. Acta Pediátr Méx.

[5] Venkatesan A, Tunkel AR, Bloch KC (2013). Case definitions, diagnostic algorithms, and priorities in encephalitis: Consensus statement of the international encephalitis consortium. Clin Infect Dis.

[6] Flores Lazo L, Olivera Ruiz R, Vences M (2022). Management of anti-NMDAR encephalitis with Rituximab: Case report in a public hospital of Lima, Peru. Rev Cuerpo Méd HNAAA.

[7] Vences MA, Saquisela VV, Barreto E, Zuñiga MA (2020). Encefalitis anti NMDAR: Reporte de caso con seguimiento a largo plazo. Rev Neuro-Psiquiatr.

[8] Suárez JM, Zurita CS, Aparicio LA, Florentín C (2022). Encefalitis autoinmune por anticuerpos contra el receptor N-metil-D-aspartato (NMDA): Serie de casos en niños. Rev Parag Reumatol.

[9] Herken J, Prüss H (2017). Red flags: Clinical signs for identifying autoimmune encephalitis in psychiatric patients. Front Psychiatry.

[10] Graus F, Titulaer MJ, Balu R (2016). A clinical approach to diagnosis of autoimmune encephalitis. Lancet Neurol.

[11] Abboud H, Probasco JC, Irani S (2021). Autoimmune encephalitis: Proposed best practice recommendations for diagnosis and acute management. J Neurol Neurosurg Psychiatry.

